# Physical Activity Interventions for Improving Cognitive Functions in Children With Autism Spectrum Disorder: Protocol for a Network Meta-Analysis of Randomized Controlled Trials

**DOI:** 10.2196/40383

**Published:** 2023-06-28

**Authors:** Longxi Li, Anni Wang, Qun Fang, Michelle E Moosbrugger

**Affiliations:** 1 Center for Leadership in Athletics University of Washington Seattle, WA United States; 2 College of Education Michigan State University East Lansing, MI United States; 3 School of Physical Education Qingdao University Qingdao China; 4 Department of Physical Education and Health Education Springfield College Springfield, MA United States

**Keywords:** children, autism spectrum disorder, physical activity, cognitive outcomes, network meta-analysis

## Abstract

**Background:**

Autism spectrum disorder (ASD) is a neurodevelopmental disorder that affects millions of children worldwide, with a current prevalence of approximately 1 in 54 children in the United States. Although the precise mechanisms underlying ASD remain unclear, research has shown that early intervention can have a significant impact on cognitive development and outcomes in children with ASD. Physical activity interventions have emerged as a promising intervention for children with ASD, but the efficacy of different types of interventions remains unclear.

**Objective:**

This study protocol aims to update the knowledge on extant literature and explore the efficacy of physical activity intervention strategies on cognitive functions in children with ASD.

**Methods:**

A systematic review and network meta-analysis (NMA) will be conducted following the PRISMA-NMA (Preferred Reporting Items for Systematic Reviews and Meta-Analyses Protocols for Network Meta-Analyses) statement. A total of 9 bibliographic databases (APA PsycInfo, CENTRAL, Dimensions, ERIC, MEDLINE Complete, PubMed, Scopus, SPORTDiscus, and Web of Science) will be systematically searched to screen eligible articles based on a series of inclusion and exclusion criteria. A study will be considered for inclusion if it is not classified as a systematic review with or without meta-analysis, was published from inception to present, includes children aged 0 to 12 years with ASD, quantitively measures cognitive outcomes, and examines treatment comprising at least 1 physical activity intervention strategy. The internal validity and quality of evidence will be evaluated using the Grading of Recommendations Assessment, Development, and Evaluation framework. Statistical analyses will be performed in the RStudio software (version 3.6; RStudio Inc) with the *BUGSnet* package and the Comprehensive Meta-Analysis software (version 3.3; Biostat Inc). The results of our NMA will be illustrated through network diagrams accompanied by geometry and league tables. Further, to rank the interventions based on their efficacy, we will use the surface under the cumulative ranking curve.

**Results:**

Our preliminary search identified 3778 potentially relevant studies. The screening of the studies based on the inclusion and exclusion criteria is ongoing, and we anticipate that the final number of eligible studies will be in the range of 30 to 50.

**Conclusions:**

This study will provide a comprehensive review of the literature on physical activity interventions for children with ASD and will use NMA to compare the efficacy of different types of interventions on cognitive outcomes. Our findings will have important implications for clinical practice and future research in this area and will contribute to the growing body of evidence supporting the use of physical activity interventions as a key component of early intervention for children with ASD.

**Trial Registration:**

PROSPERO CRD42021279054; https://www.crd.york.ac.uk/prospero/display_record.php?RecordID=279054

**International Registered Report Identifier (IRRID):**

DERR1-10.2196/40383

## Introduction

### Background

Autism spectrum disorder (ASD) is a lifelong developmental disorder. Individuals with ASD commonly present symptoms of insufficient social interaction and communication and repeated and restricted behavior patterns that have a negative impact on daily functions. The lack of adapting, planning, and organizing skills has a negative influence on personal, physical, mental, academic, vocational, and social domains. The onset of ASD typically presents during the ages of 1 to 2 years, whereas some symptoms may be observed during the first year after birth [1]. Some categories of ASD have been specified such as Asperger syndrome, childhood disintegrative disorder, and pervasive developmental disorder [2]. The World Health Organization has indicated that children with ASD exhibit deficits in cognitive functions, characterized by social impairment, abnormalities, and repetitive stereotypic behavior [3,4]. Approximately 17% of children were diagnosed with a range of complex neurodevelopmental disabilities between the ages of 3 and 17 years; 1 in 54 children was diagnosed with ASD [5,6]. The diagnosis rate in children has continuously increased, moving from 1.1% to 2.5% in the past decades [7,8]. Although the prevalence of diagnoses is increasing, many evidence-based interventions are still in their developmental stages. Specifically, the effectiveness of physical activity interventions in children with ASD is not yet fully understood.

Childhood is a critical period for neurodevelopment, especially for the development of children’s memory, attention, creativity, motor skills, and executive functions (EFs) [9]. ASD is a complex neurodevelopmental disorder, and its prognosis is impacted by environmental, genetic, and physiological factors [1]. Several neurotransmitters, including brain-derived neurotrophic factors, which contribute to brain development, demonstrate correlations with ASD and other neurodevelopmental impairments [10]. The connectivity of the frontal cortex and prefrontal cortex with other parts of the brain was found to be related to ASD behavioral symptoms [11-13]. High risk for an atypical development of the ability to recognize and attribute mental states was found in the autism population [14]. Language and joint attention for social functioning and cognitive control behaviors have been shown to be linked to changes in the early maturation of the brain [9,14-16]. An imbalanced process of integrating information such as contextual or sensory perception might be related to ASD symptoms [17].

Cognitive function is manifested as the ability to process thoughts, such as learning and solving problems [18]. Cognitive function has been categorized into EFs and non-EFs. EFs present an individual’s adaptive skills and ability to conduct goal-oriented meaningful actions [19]. Non-EFs include complex attention, learning and memory, visual function, language function, and social function [1]. Solomon et al [20] indicated that EF hypothesis is an influential cognitive theory of ASD, which proposes that “deficits in planning, inhibitory control, attentional set shifting and working memory are central to the disorder.” Social and communication skills are part of the characteristics of cognitive function, which individuals with ASD usually lack, and are also impacted by the age and development window [21]. In children, cognitive function develops along with the human brain [22]. An example of proneurogenic modulators is exercise, which increases the level of brain-derived neurotrophic factors, endocannabinoids, and microbiota diversity, leading to hippocampal neurogenesis and long-term changes in cognitive function [23,24]. Various interventions for children with ASD have been studied, such as music therapy, which has been shown to improve social interaction in preschool children with ASD [14]. In addition, physical activity is an effective approach to improve cardiovascular fitness and cognitive function in children and adolescents [25].

Evidence shows that physical training positively impacts the cognitive function of adults and their offspring, which is encouraging for investigations on the effect of physical activity on youth [26]. In this study, we expect to use a statistical methodology to explore the effectiveness of different types of physical activity intervention strategies (PAIS) on cognitive function in children. We believe that this study is a promising starting point to guide future health-related practices in youth. Closely related to the release of dopamine in the human body, physical activities have also been proven to provide emotional support to children, such as by lowering levels of anxiety or depression [27]. Promising evidence has been accumulated of physical activity leading to improvements in cognition, including improvements in both academic and behavioral performances in children with ASD [3,28-31]. Liang et al [32] reported that chronic exercise interventions are beneficial for overall EFs, especially for cognitive flexibility and inhibitory control in children and adolescents with ASD. Several studies have indicated a significant increase in social engagement in children with ASD after they participated in physical activities, especially in group activities [33-35]. Furthermore, physical activity proved beneficial in reducing stereotypical behaviors within the population of children with ASD [36].

In prior research, various types of PAIS were investigated individually or through narrow groups. In this study, a full range of PAIS will be examined. PAIS include aerobic exercises, mindfulness practices, school physical education programs, and Montessori methods [32,37]. Fang et al [38] concluded favorable results for the effect of exergaming interventions on cognitive functions, but synthesis results from a follow-up meta-analysis are lacking. Horseback riding was found to be beneficial for cognitive functioning, social interaction, and sensory processing improvement in children with ASD [33,35]. Some other physical activities, such as yoga and dance, were also introduced as interventions for children with ASD, which led to positive outcomes in behavioral functioning [39]. The clinical conditions of participants, as well as the session frequencies and lengths of the interventions, need to be researched more. Hence, there is an urgent need for researchers to explicitly examine what type of PAIS is the most beneficial for children with ASD.

Systematic reviews and pairwise meta-analyses cannot be used to compare treatment effects across interventions and indirect effects. Therefore, the network meta-analysis (NMA) is proposed as a novel analysis technique in the field of physical activity and health promotion to fill this gap and satisfy the urgent need [40]. To the best of our knowledge, this will be the first NMA applied to systematically review and analyze the efficacy of PAIS on cognitive functions in children with ASD. Therefore, our expectation is to explore PAIS based on selected direct and indirect head-to-head trials to suggest important considerations for decision-making in the field of pediatric health promotion.

### Mini Review

#### Overview

To gain a comprehensive overview of the existing literature as well as justify the necessity of conducting an NMA on PAIS for children and adolescents with ASD, we conducted a mini review based on the systematic reviews and meta-analyses in the current literature. The literature search was conducted on PubMed, APA PsycInfo, SPORTDiscus, Web of Science, CENTRAL, ERIC, SocINDEX, MEDLINE Complete, and Dimensions. The keywords used for the literature search involved 4 domains: intervention mode, cognitive performance, population, and autism. Combinations of the keywords regarding each domain were “physical activity OR PA OR exercise OR sport*” AND “cognitive function OR cognition OR executive function OR inhibitory control OR memory OR communication OR stereotyp* behavior*” AND “children OR kid* OR teenager OR students” AND “autism spectrum disorder OR ASD.” Eligible studies should meet the following criteria: (1) systematic review or meta-analysis written in English and published between 2001 and 2021, (2) inclusion of children or adolescents with ASD, (3) review of PAIS, and (4) examination of the effects of PAIS on cognitive functions.

#### Literature Search

**Figure 1:**
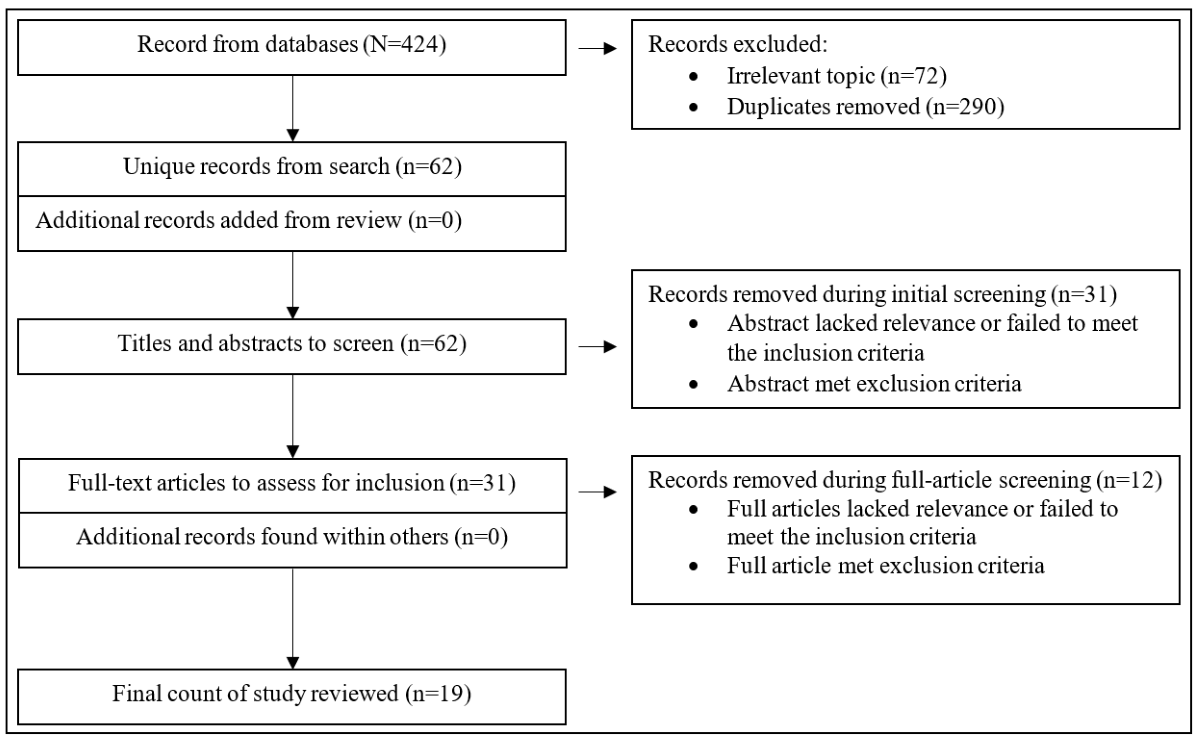
Flowchart of searching and screening in mini review.

Two authors (LL and AW) independently conducted the search and screening process. The initial search resulted in 424 studies. Of these 424 studies, after the removal of 362 (85.4%) duplicate and irrelevant items, 62 (14.6%) items remained for the next phase of screening. Of these 62 articles, 31 (50%) articles were excluded based on abstract examination, leaving 31 (50%) articles for full-text review. Of these 31 articles, the final step of screening removed 12 (39%) articles because of the following reasons: no cognitive outcomes were measured (n=10, 32%) and populations other than children or adolescents were included (n=2, 6%). Therefore, a total of 19 (61%) of the 31 systematic reviews with or without meta-analysis met the inclusion criteria. The process of study selection is presented in Figure 1.

#### Characteristics of the Included Studies

The median publication year of these studies was 2019 (range 2008-2021), which implies a rapid increase in the number of published review papers on the relevant topic in recent years. The number of studies included in each review ranged from 7 to 41. A variety of types of the included review papers was identified, such as systematic review (9/19, 47%), meta-analysis (7/19, 37%), and systematic review with meta-analysis (3/19, 16%). In addition to individuals with ASD, the included review studies also examined individuals with cognitive impairment due to one of the following reasons: attention-deficit/hyperactivity disorder, cerebral palsy, developmental delay, developmental coordination disorder, and Down syndrome. Common symptoms observed in individuals with these disorders are difficulties in paying attention, performing tasks, and engaging in social interaction [1]. Most studies focused on children and adolescents [14,32,34,41-51], whereas Fang et al [38], Fragala-Pinkham et al [52], Healy et al [53], Lang et al [54], Ruggeri et al [55], and Sowa and Meulenbroek [56] conducted studies involving both children and adult participants. Experimental groups were compared with their counterparts, that is, active behavioral control groups, waiting list control groups, or standard care groups. The study characteristics are illustrated in Table 1.

The PAIS applied in the intervention arms typically consisted of two or three 30- to 60-minute sessions per week for 8 to 16 weeks. Overall, 3 (16%) of the 19 studies included only randomized controlled trials (RCTs) [14,47,50], and others (16/19, 84%) partially included quasi-experimental designs with controlled trials [32,41-44,48,49,51,53-56], cohort study [34], pre- and posttest and case-control studies [38,45,52], and descriptive study [46]. Moreover, 1 (5%) of the 19 studies lacked a clear definition of the age range of children and a discussion of the risk of bias (RoB) [41]. The interventions implemented in the included studies were conducted through various types of physical activities. Physical exercise– and motor skill–related interventions, such as aerobic exercise, resistance training, and aquatic activities, were mostly adopted in the clinical practice [32,34,41-56]. In addition, 2 (11%) of the 19 studies investigated effects of exergaming and technology interventions for individuals with ASD [38,43].

The included studies investigated the effects of physical activity interventions on cognitive functions, with small to moderate effect sizes on the improved global EF [32,50]. Specifically, the included reviews reported enhanced performance in terms of working memory, attention, task switching, and inhibitory control [34,50]. The evidence also indicated the beneficial effects of social functioning and communication skills, which can be attributed to the group-based activity performed during the interventions [42,44]. Learning and memory outcomes due to PAIS were not fully explored in the selected studies. The other limitation of the included systematic review and meta-analysis studies was the combination of children and adolescent groups. However, age was recognized as a significant confounder in ASD research. Although common clinical treatments, including educational, family, speech, and occupational therapies, showed favorable effects, widely supportive evidence showed that physical activity was beneficial for children’s lifelong cognitive and motor development. Therefore, it is necessary to explore the effects of PAIS treatment on children’s cognitive outcomes from the perspective of physical activity and health promotion. Results are encouraging because various types of evidence showed that PAIS were effective in enhancing cognitive function in children with ASD; the foundation for the future in-depth data analysis has been established.

**Table 1:** Characteristics of the studies included in the mini review.

Study	Study type	Participant, n	Study population	Intervention used (intervention arm)	Outcome measures^a^	Main outcomes
Aleksandrovic et al [41], 2016	SR^b^	13	Children (aged <18 years and with ASD^c^)	Aquatic activities (Armbruster or Halliwick methods or the constant time delay procedure for learning swimming skills and social and floor warm-up activities)	MS^d^ (ABC-2^e^, WOTA^f^, HAAR^g^, YMCA^h^ checklist, and Aquatic Skills Assessment)	Aquatic skills were effectively improved
Bremer et al [34], 2016	SR	13	Children and youth (aged ≤16 years; at least one participant with a diagnosis of ASD or PDD^i^)	Physical exercise intervention (jogging, swimming, yoga or dance, horseback riding, and martial arts)	CF^j^+SF^k^ (GARS-2^l^, SRS^m^, sensory profile, ABC-C^n^, ABC^o^, VABS-2^p^, frequency of child specific behaviors, BOSS^q^, SSBS-2^r^, and BASC-2^s^)	Improvements in stereotyped behaviors, social behavior cognition, and attention
Cameron et al [42], 2020	SR	17	Children (aged 3-6 years) with autism, CP^t^, Down syndrome, DCD^u^, DD^v^	Fundamental motor skill group intervention by primary researcher	MS (PDMS-2^w^: GMQ^x^)	Results were inconclusive to support motor-based interventions in this age group owing to variability in intervention types
Case and Yun [43], 2019	MA^y^	18	Preschool (aged 3-5 years) and school-aged children (aged 6-17 years) with ASD	FMS^z^ (direct instruction activities), PA^aa^ (whole body movement and coordination activities), technology (eg, video games), EAT^ab^ (eg, horseback riding)-based interventions	MS (PDMS, PDMS-2, TGMD^ac^, TGMD-2, TGMD-3 BOT-2^ad^, M-ABC^ae^, and M-ABC-2)	Overall effect size on gross motor outcomes (δ=0.99; *P*<.001), FMS (*g*=0.68; *P*<.001), PA (*g*=1.20; *P*=.003), technology (*g*=1.42; *P*=.21), and EAT (*g*=1.20; *P*=.005)
Chan et al [44], 2021	MA	12	Children and adolescents (aged <18 years and with ASD)	Individual, combined, or group-based PA (eg, Karate, horseback riding, aquatic, mind-body, football, outdoor adventure, or active recreation)	SF (GARS-2, SRS, VABS, ATEC^af^, and SSIS^ag^)	COM^ah^ (SMC^ai^=.27, 95% CI 0.06 to 0.48) and social functioning (SMC=.39, 95% CI 0.15 to 0.63)
Fang et al [38], 2019	SR	10	Children and young adults (aged 5 to 21 years and with ASD)	Exergaming (eg, platforms and games included DDR^aj^, cybercycling, FroggyBobby, Xbox Kinect, Nintendo Wii, and Makoto Arena)	CF+MS (physical outcomes [eg, BMI and jump distance] and cognitive outcomes [eg, BRIEF^ak^])	Favorable results for the effect of exergaming interventions on participants’ physical and cognitive functions
Ferreira et al [45], 2019	SR and MA	8 (SR) + 8 (MA)	Children and youngsters (aged <16 years and with ASD)	Aerobic exercise, Kata techniques training, and ball exercise	CF (GARS-2, SSB^al^, and stereotypic behavior observations)	PA was effective in reducing stereotypic behavior (SMD^am^=1.11; *P*=.009)
Fragala-Pinkham et al [52], 2021	SR	8	Children and young adults (aged 7 to 26 years and with ASD, Down syndrome, or UID^an^)	Lower extremity cycling (eg, learning to ride a 2-wheeled bicycle, stationary cycling, and CO-OP^ao^ approach)	CF+MS (eg, cycling time and distance, cycling skill checklist, KT^ap^, KTFist^aq^, TOLT^ar^, and PPT-C^as^)	Weak evidence and heterogeneous results regarding MS and CF in children and young adults with intellectual disabilities between cycling and control groups
Healy et al [53], 2018	MA	29	Youth (aged 2 to 22 years and with ASD)	Recreational activities in APE^at^, PE^au^, and other settings (eg, yoga, kata program, aquatic skill program, dance, mind-body exercise, and a combination of all activities)	CF+MS (psychometric tools or scales were not specifically discussed)	Overall positive moderate effect exhibited (*g*=0.62; *P*<.001); cognitive function (*g*=0.28, 95% CI −1.19 to 1.74), psychomotor function (*g*=1.21, 95% CI 0.41 to 1.66)
Howells et al [46], 2019	SR and MA	11 (SR) + 7 (MA)	Children (aged 5 to 12.9 years and with ASD)	Individual and group PA (eg, included a range of blocking, punching, sticking, and kicking techniques; climbing rope ladder or rope elevator; horse riding; group sport games)	SF+COM (VABS, SRS, BASC-T, and GARS-2)	Nonsignificant effect for communication (*g*=0.13, 95% CI −0.12 to 0.38) and a significant improvement in overall social functioning (*g*=0.45, 95% CI 0.19 to 0.72)
Huang et al [47], 2020	MA	12	Children and adolescents (aged 3 to 18 years and with ASD)	PA interventions (eg, Kata techniques, therapeutic horseback, outdoor activity, karate, aquatic sport, trampoline, table tennis, Tai Chi, and exergaming)	CF+MS+SF+COM (GARS-2, BOT-2, SRS, PPVT-4^av^, SALT^aw^, ATEC, ABC, MABC-2^ax^, CBS^ay^, and WCST^az^)	Significant positive effects on social interaction (SMD=−0.58, 95% CI −0.87 to −0.29), COM (SMD=−0.29, 95% CI −0.55 to −0.04), stereotyped behavior (SMD=−0.13, 95% CI −0.46 to 0.20), and motor skills (SMD=1.02, 95% CI 0.33 to 1.71)
Lang et al [54], 2010	SR	18	Children, adolescents, and adults (aged 3 to 41 years and with ASD)	PA (eg, walking, jogging, playing catch with a ball, riding a stationary bike, swimming, and weight training)	CF+PF (psychometric tools or scales were not specifically discussed)	Exercise decreased stereotypy, aggression, off-task behavior, and elopement
Liang et al [32], 2021	SR and MA	14 (SR) + 7 (MA)	Children (aged 5 to 17 years and with ASD)	Physical activity or exercise on EFs^ba^ (exergaming, jogging, table tennis, progressive muscle relaxation, martial arts, basketball, etc)	CF (BRIEF, Stroop, CTT^bb^, HKLLT^bc^, DSFBT^bd^, FPT^be^, TOLT, GNG^bf^, CBTT^bg^, Hearts and Flowers test, and attention sustained test)	Positive effect on global EFs (*g*=0.34, 95% CI 0.08 to 0.60), CF (*g*=0.31, 95% CI 0.05 to 0.57), IC^bh^ (*g*=0.49, 95% CI 0.19 to 0.80]), and WM^bi^ (*g*=0.21, 95% CI −0.09 to 0.51)
Petrus et al [48], 2008	SR	7	Children (aged 4 to 15 years and with ASD)	PA or exercise (eg, aquatic, jogging, ball playing, and aerobic exercise)	CF+MS (stereotypical behaviors and MS were observational measured in frequency, duration, or counts)	Exercise might reduce stereotypic behaviors in the short term
Ruggeri et al [55], 2020	SR	41	Children and adolescents (aged 3 to 19 years and with ASD)	Motor and physical activity (eg, soccer, exergaming, throwing, aquatic, fitness or strength, horse riding, hippotherapy, stationary biking, gymnastic, and indoor climbing programs)	MS+ML (eg, VABS-2, BOT, BOT-2, MABC-2, PDMS-2, TGMD-2, TGMD-3, m-PEDI^bj^, ASC^bk^, YMCA checklist, HAAR, SCS^bl^, and GAS^bm^)	Motor skill and learning, body structure, and function outcomes were improved with various PA interventions
Semple [49], 2019	SR	8	Youth (aged 3 to 18 years and with ASD)	Yoga, mindfulness-based interventions, Qi Gong, and meditation	CF+MS+SF+COM (psychometric tools or scales were not specifically discussed)	Improvements in prosocial behaviors, social cognition, communication, motivation, and motor control via yoga and mindfulness-based interventions
Sowa and Meulenbroek [56], 2012	MA	16	Children, adolescents, and adults (aged 4 to 41.3 years and with ASD)	Individual and group PA interventions (eg, jogging, therapeutic horseback rides, bike riding, walking, stationary cycling, aquatic exercising, and swimming)	MS+SF+COM (psychometric tools or scales were not specifically discussed)	Individual programs exhibited positive effects on motor skills (*r*=−0.32; *P*=.004) and social interactions (*r*=−0.62; *P*<.001) compared with group programs
Zhang et al [50], 2020	MA	11	Children (age: N/A^bn^) with ADHD^bo^ or ASD	Chronic PA intervention (eg, rhythm, integrated PA, modified aerobic games, exergaming, and taekwondo)	CF+MS (BOT-2, WCST, TGMD-2, Stroop test, Flanker task, CBTT, etc)	Significant improvement in overall EF, IC, cognitive flexibility, and gross MS (SMD ranged from 0.85 to 1.30); no significant improvements in working memory and fine MS
Teh et al [51], 2021	MA	22	Children (N/A with ASD)	Physical exercise interventions (jogging, cycling, exergaming, ball exercise, therapeutic horse riding, martial arts)	CF (GARS-2, ATEC, ABC, BASC-2, observational measures or time intervals on targeted behaviors, etc)	Large overall ES^bp^ (*g*=1.16) on reducing stereotyped motor behaviors across participants, treatment, and levels from a multilevel modeling MA

^a^Only autism spectrum disorder–relative interventions and outcomes are summarized because of the established inclusion criteria of this study.

^b^SR: systematic review.

^c^ASD: autism spectrum disorder.

^d^MS: motor skills.

^e^ABC-2: Assessment Battery for Children, Second Edition.

^f^WOTA: Water Orientation Test Alyn.

^g^HAAR: Humphries Assessment of Aquatic Readiness.

^h^YMCA: Young Men’s Christian Association.

^i^PDD: pervasive developmental disorder.

^j^CF: cognitive flexibility.

^k^SF: social functioning.

^l^GARS-2: Gilliam Autism Rating Scale, Second Edition.

^m^SRS: Social Responsiveness Scale.

^n^ABC-C: Aberrant Behavior Checklist–Community.

^o^ABC: Aberrant Behavior Checklist.

^p^VABS-2: Vineland Adaptive Behavior Scales, Interview Edition.

^q^BOSS: Behavioral Observation of Students in Schools.

^r^SSBS-2: School Social Behavior Scales, Second Edition.

^s^BASC-2: Behavioral Assessment System for Children, Second Edition.

^t^CP: cerebral palsy.

^u^DCD: developmental coordination disorder.

^v^DD: developmental delay.

^w^PDMS-2: Peabody Developmental Motor Scales, Second Edition.

^x^GMQ: gross motor quotient.

^y^MA: meta-analysis.

^z^FMS: fundamental motor skills.

^aa^PA: physical activity.

^ab^EAT: equestrian-assisted training.

^ac^TGMD: Test of Gross Motor Development.

^ad^BOT-2: Bruininks-Oseretsky Test of Motor Proficiency, Second Edition.

^ae^M-ABC: Movement Assessment Battery for Children.

^af^ATEC: Autism Treatment Evaluation Checklist.

^ag^SSIS: Social Skills Improvement System.

^ah^COM: communication.

^ai^SMC: standardized mean change.

^aj^DDR: Dance Dance Revolution.

^ak^BRIEF1: Behavior Rating Inventory of Executive Function.

^al^SSB: Self-stimulatory Behaviors.

^am^SMD: standardized mean difference.

^an^UID: unspecified intellectual disability.

^ao^CO-OP: Cognitive Orientation to daily Occupational Performance.

^ap^KT: Knock-Tap.

^aq^KTFist: Knock-Tap-Fist.

^ar^TOLT: Tower of London Test.

^as^PPT-C: Purdue Pegboard Test–Combined.

^at^APE: adapted physical education.

^au^PE: physical education.

^av^PPVT-4: Peabody Picture Vocabulary Test, Fourth Edition.

^aw^SALT: Systematic Analysis of Language Transcripts.

^ax^MABC-2: Movement Assessment Battery for Children - Second Edition.

^ay^CBS: Clancy Behavior Scale.

^az^WCST: Wisconsin Card Sorting Test.

^ba^EF: executive function.

^bb^CTT: Color Trails Test.

^bc^HKLLT: Hong Kong List Learning Test.

^bd^DSFBT: digit span forward and backward test.

^be^FPT: Five-Point Test.

^bf^GNG: Go-No-Go task.

^bg^CBTT: Corsi Block-Tapping Task.

^bh^IC: inhibitor control.

^bi^WM: working memory.

^bj^m-PEDI: mobility scale of the Pediatric Evaluation of Disability Inventory.

^bk^ASC: Aquatic Skills Checklist.

^bl^SCS: Swimming Classification Scale.

^bm^GAS: Goal Attainment Scaling.

^bn^Criteria of age was not mentioned in search strategy.

^bo^ADHD: attention-deficit/hyperactivity disorder.

^bp^ES: effect size.

#### The Present NMA Protocol

An insight into the direction of future research was provided based on the preliminary findings of the mini review. Previous outcomes suggested that varying types of PAIS were effective in improving cognitive functions and motor performance, reducing frequency of the symptoms, and enhancing social and communication skill development in children with ASD and other cognitive impairments. The quality of evidence is concerning because of the limited number of meta-analyses on RCTs [53]; we expected to simply include true experimental designs (eg, RCTs), which might ensure robust synthesis results and increase the power to detect the effects of interventions [51,57]. The age groups were heterogeneous in nature, whereas we expected to focus particularly on children and explore the efficacy of PAIS on their cognitive performances. However, the limitation of the pairwise meta-analysis procedure was that it was only appropriate for discerning outcome effects “between a selected intervention strategy and a selected comparison or control condition” [40]. Whereas pairwise meta-analyses only allowed the comparison of multiple outcomes from a single treatment, NMA would allow the comparison of the efficacies of multiple PAIS on multiple outcomes [58]. Concomitantly, there is a need to conduct an NMA to obtain a more accurate estimation of the efficacy of PAIS in the population. We aim to provide an updated review of the extant literature in the field of physical activity and health promotion using an appropriate network meta-analytic model. Therefore, the following protocol was developed based on the findings and limitations identified in the mini review.

## Methods

### Overview

This review protocol has been registered in PROSPERO (CRD42021279054). The NMA will comply with the PRISMA-NMA (Preferred Reporting Items for Systematic Reviews and Meta-Analyses Protocols for Network Meta-Analyses) statement [59,60]. A component-based Bayesian framework NMA will be conducted following the procedures described in the subsequent sections [61].

### Search Strategy

The following 9 bibliographic databases will be searched: APA PsycInfo, CENTRAL, Dimensions, ERIC, MEDLINE Complete, PubMed, Scopus, SPORTDiscus, and Web of Science. In general, the search will be limited to RCTs in English published from inception to present. Keywords combinations and MeSH (Medical Subject Headings) terms used in the search are listed in Table 2.

**Table 2:** Flowchart of search strategy.^a^

Concept	Keywords
1. Outcome: cognitive functions	“Cognitive function*” or “cognitive outcomes” or executive function* or “neurocognitive tasks” or questionnaires or inventory or task* or restricted repertoire or autism spectrum disorder (MeSH^b^ terms) or children behavior disorder (MeSH terms) or autistic disorder (MeSH terms) or stereotypic movement (MeSH terms) or motor skills disorder (MeSH terms) or cognition (MeSH terms) or communication disorder (MeSH terms) or learning disabilities (MeSH terms) or DSM-V^c^ (MeSH terms)
2. Participants: children and adolescents	Children or adolescent* or young people or school-aged or teen or young adult* or youth or kids or teenager* Exclude (NOT): smoking or drinking or criminal* or injury or injuries or accident* or trauma or adult* or older adult* or elderly or seniors or geriatric*
3. Exposure: comparator	Control* or “control group” or subject* or “human subjects” or human-related or waiting list
4. Exposure: exercise types	“Physical activity” or intervention* or exercise* or fitness or “physical exercise” or sport* or psychomotor or motor activity or recreational sport* or school-based or computerized training or hybrid or games or aerobic or “martial arts” or Tai Chi or “mindfulness practice” or classroom curricula or Montessori methods or “resistance training”
5. Design	Experimental* or clinical trial or randomized control trial* or RCT^d^ or nonrandomized comparison or NRS^e^ Exclude (NOT): observational studies or cross-sectional or case control or cohort or systematic review or meta-analysis or report or protocol or qualitative or mixed method

^a^The bibliographic databases will be searched including PubMed, SPORTDiscus, APA PsycInfo, MEDLINE Complete, ERIC, Scopus, Web of Science, CENTRAL, and Dimensions. We will use “OR” to separate keywords within each concept and “AND” to separate each concept. MeSH terms will be used for PubMed search only. Google Scholar will be used for cross validation for search results from multiple bibliographic databases.

^b^MeSH: Medical Subject Headings.

^c^DSM-V: Diagnostic and Statistical Manual of Mental Disorders, Fifth Edition.

^d^RCT: randomized controlled trial.

^e^NRS: nonrandomized studies.

### Inclusion and Exclusion Criteria

The following subsections on population, intervention, comparator, outcomes, and study design explicate the various elements of Population, Intervention, Comparison, Outcome Measures, and Design (PICOS), which is the framework used in this study to facilitate the literature search [58].

#### Population

Studies including children aged between 0 and 12 years with a diagnosis of ASD (Asperger syndrome, autism, or unspecified developmental disorder) with varying severity (mild, moderate, or severe) will be accepted. The study should include at least 1 diagnostic criterion regarding the Diagnostic and Statistical Manual of Mental Disorder [1], International Statistical Classification of Diseases and Related Health Problems, or Autism Diagnostic Observation Schedule. In addition, demographic information, including age, gender, and sample size, should be reported.

#### Intervention

Studies intended to increase cognitive functions in participants with ASD using physical activity or exercise interventions will be included. Studies evaluating physical activity exhibited in school-based physical education, exergaming, mindfulness practice (eg, yoga, martial arts, and Tai Chi), aerobic exercise and sports, and any types of therapeutic physical activity interventions will be included.

#### Comparison

Studies with comparators such as active behavioral control group, waiting list control group, and standard care group will be eligible for inclusion in the network of evidence.

#### Outcome Measures

Studies should report mean differences in cognitive function measurements between the beginning of the intervention and follow-up test. Studies should have obtained at least 1 validated outcome measurement of cognitive functions. Study features must include the measurement tool with either objective or self-reported patterns.

#### Design

RCTs should have assessed the outcome measures before (baseline) and after the intervention; the study should have included >1 session of intervention.

#### Exclusion Criteria

The following studies were excluded: studies including participants who were aged >12 years or were unable to conduct psychomotor movements, studies not involving an RCT, studies where the intervention lasted <8 weeks, and studies not written in English.

### Study Selection and Data Extraction

**Figure 2:**
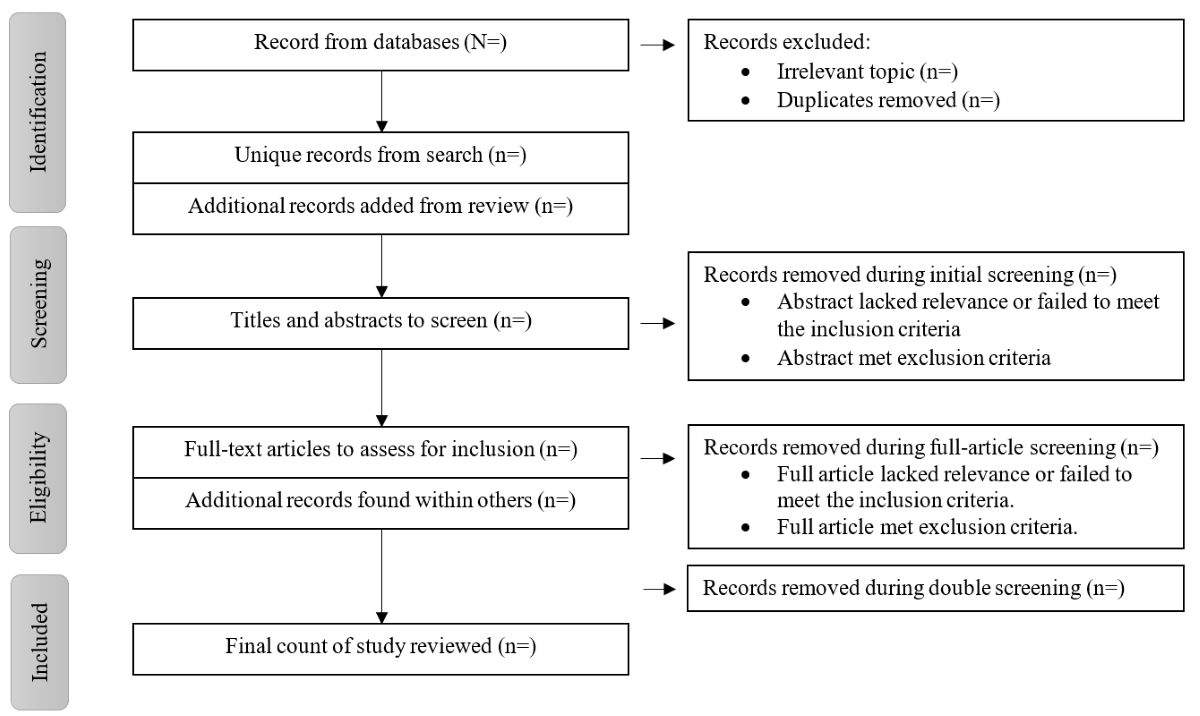
PRISMA (Preferred Reporting Items for Systematic Reviews and Meta-Analyses) flowchart of searching and screening.

Recording and managing related literature will be accomplished using the Mendeley software (Mendeley Ltd). Two authors will independently screen the titles and abstracts of the searched peer-reviewed studies based on the preestablished criteria for inclusion. A third reviewer will be involved to initiate a full-text evaluation if any conflicted selections occur. The PRISMA (Preferred Reporting Items for Systematic Reviews and Meta-Analyses) flowchart illustrates the search and screening processes (Figure 2).

Study characteristics, including author information, publication year, mean age in sample, diagnostic criteria for ASD, study period, intervention arms, and study design, will be summarized using Microsoft Excel (version 2010; Microsoft Corp). Specifically, we will adopt a thematic organization approach to categorize intervention arms within all relevant RCTs into six predefined categories: (1) school physical education programs, (2) exergaming, (3) aerobic exercise and sports, (4) mindfulness practice, (5) computerized and noncomputer games combination, and (6) therapeutic physical activity interventions [32,37]. The project administrator will serve as the coordinator, dealing with potential disagreements or unclearly defined PAIS categories. Internal discussions and consultation with an experienced researcher in physical activity and health promotion are considered 2 solutions for disagreements and uncertainties. The interrater agreement rate, Cohen *k*, between reviewers regarding the eligibility criteria of the study will be reported [62]. Note that grouped and ungrouped or supervised and unsupervised intervention arms will be separated as 1 effect size per treatment [51]. Quantified outcomes (mean of the intervention arms and comparators, SDs, and 95% CIs) will be extracted from the aforementioned categories, including measurement results regarding cognitive functions (eg, global EFs, working memory, thinking flexibility, inhibitory control, attention, or memory outcomes). In the NMA, we will compare the effects of the predefined intervention categories on participants’ cognitive performance. Raw data (eg, sample sizes and *t*, *F*, or *P* values) will be extracted to help estimate the effect size if the means and SDs were not reported [51]. When necessary, the corresponding authors of selected studies will be contacted to obtain unpublished and missing data to allow for effect size estimates.

### Assessment of RoB

The Grading of Recommendations Assessment, Development, and Evaluation will be used to assess the quality of the selected studies in the NMA and pairwise meta-analysis [63-65]. Two authors will independently assess bias due to randomization, deviations from intervention, missing data, outcome measurement, and the selection of reported results in the selected RCTs. Each item is classified as high, some concerns, or less or unclear or no information upon RoB, and the results of the grading of evidence quality will then be generated as a table (Cochrane RoB tool; version 2.0 [66]). Homogeneity will be assessed by summarizing the selected study characteristics in a general table and observing whether the treatments and participant characteristics are suitable and comparable across all studies [40,58]. Consistency should be ensured such that direct evidence and indirect evidence essentially agree with each other without any discrepancies [40]. The R package (R Foundation for Statistical Computing) developed by van Valkenhoef et al [67] will be used to evaluate the inconsistency between indirect and direct comparisons via the node-splitting method. We evaluated consistency using a node-splitting technique that compares the direct and indirect estimates for each comparison. Transitivity will be qualitatively examined to ensure that direct trials do not differ with respect to the distribution of effect modifiers in which all treatments are jointly randomizable [68].

### Statistical Analysis

The primary outcome for this study will be changes in cognitive functions. However, unlike physiological and biochemical indexes, cognitive performances were measured through preestablished validated psychometric instruments (illustrated in Table 1). Guiding through the approach applied by Désaméricq et al [69], the effect size will be calculated for each extracted result, and these effect sizes will then be combined into 3 cognitive scores, as listed in Multimedia Appendix 1. An omnibus composite unit free score of cognitive outcomes will be created by averaging all subordinate domain effect sizes within a study per intervention arm. Studies including multiple treatments or outcomes will provide the effect size of each treatment or outcome. Extracted data, such as sample size, treatment and control means and SDs for between-group designs, and preintervention and postintervention mean and SDs for within-group designs, will be converted to correlated summary results, standardized mean differences (SMDs), for NMA to explore the efficacy of PAIS on cognitive performance [70]. The effect sizes will be computed using SMD with 95% CIs between groups [32,71]. Hedges *g* in a random model will be used in consideration of potential variances across the selected studies if the number of studies is <20 [32,51,72]. Effect sizes will be classified as small, moderate, or high, respectively, with regard to the cutoff values of 0.2, 0.5, and 0.8 [32,73]. The calculations will be computed through a between-case SMD estimator, the web application *scdhlm* [74]. Heterogeneity will be evaluated through the means of *Q* test and *I*^2^ values, considering statistical heterogeneity of 25% as low, 50% as medium, and 75% as high [32,75,76]. Sensitivity analyses will be conducted to determine whether any study separately influenced the overall results [77]. To minimize the impact of heterogeneous outcomes or extreme values, we will apply the threshold of *z* score 3.29 to screen out outliers in the mean effect scores [46,53,78]. The level of significance will be set at.05 in this study. The NMA will be performed in RStudio (version 3.6; RStudio Inc [79]) using the *BUGSnet* package (version 1.1.0 [80]).

### Moderation Analysis

Moderation analysis will be conducted to examine the variability among potential sources owing to heterogeneous PAIS settings. Moderators such as participant age, exercise intensity, and intervention setting (eg, group vs individual) will be examined using robust variance estimation [51]. Our expectation is to provide another perspective of synthesis results to elicit the higher order outcomes from NMA. Moderation analysis will be performed in the Comprehensive Meta-Analysis software (version 3.3; Biostat Inc).

### Ethical Considerations and Dissemination

The ethics approval process is exempted because this study will be based on findings of previous studies. This review protocol has been registered in PROSPERO with the aims of promoting transparency and minimizing the RoB [81]. Future results will be submitted to a peer-reviewed journal of the relevant field for consideration for publication.

### Expected Findings

We expect to compare the effects of different PAIS on cognitive outcomes (overall and by domain) over a period of treatment (at least 8 weeks). We will perform an NMA; we will select all RCTs that consider PAIS as a treatment for cognitive outcomes in children with ASD. This study will bridge the gap and provide a novel perspective and robust evidence to clinicians and practitioners for future decision-making in children with ASD. Forecasted results will include study characteristics, qualitative appraisal of cognitive tasks and intervention technologies, and quality of the selected studies; visual demonstration of synthesis results such as network diagrams and associated geometry will be generated and evaluated, respectively. Expected results will be able to rank the efficacy of multiple PAIS (eg, aerobic exercise, mindlessness practices, school-based physical education programs, and exergaming) with effect size differences demonstrated between multiple treatments. In addition to network plots, results will be illustrated in tables of network characteristics, data plots, league tables and league heat plots, surface under the cumulative ranking curve plots, and rankograms. An in-depth discussion will be held. In general, results may reveal the rank efficacy of PAIS, in combination or as single treatment, on promoting cognitive functions and further social and communication skills in children with ASD. In addition, owing to the COVID-19 pandemic, social restrictions and isolation were enforced, which led to an increase in sedentary behavior and physical inactivity, which, in turn, increased the risks of cardiovascular disease and mental wellness crisis in children [82-84]. There are more concerns especially regarding children with ASD [85,86]. We will include an in-depth discussion of feasible interventions along with safety suggestions for programming during the global pandemic.

## Results

Our preliminary search identified 3778 potentially relevant studies. The screening of the studies based on the inclusion and exclusion criteria is ongoing, and we anticipate that the final number of eligible studies will be in the range of 30 to 50.

## Discussion

### Principal Findings

With the described protocol, we aim to address the urgent need for effective PAIS to reduce the negative impact on cognitive outcomes in children with disabilities, including those with ASD. As noted in our mini review, research suggests that PAIS may have a positive impact on cognitive function in children with ASD, but there is limited information on the comparative effectiveness of different types of PAIS. Therefore, our study represents an important contribution to the field, as it is the first systematic review and NMA protocol to propose to examine the efficacy of PAIS on cognitive performance in children with ASD.

Preliminary results from the mini review suggested that several types of PAIS, including yoga, martial arts, and aerobic exercise, may have positive effects on cognitive performance in children with ASD. Specifically, we found that these interventions were associated with improvements in cognitive flexibility, attention, and memory. Notably, our NMA will allow us to indirectly compare the effectiveness of different types of PAIS, providing valuable insights into which interventions may be most effective for improving cognitive function in this population.

Our study represents an important contribution to the literature on the efficacy of PAIS in improving cognitive performance in children with ASD. Although we have taken steps to minimize bias and ensure the quality of the study, as listed in Multimedia Appendix 2, there are some limitations that should be considered when interpreting our results. First, our study is limited by the quality and heterogeneity of the included studies. Although we have conducted a comprehensive search of multiple databases and used rigorous inclusion criteria, some relevant studies may have been missed. Second, as with any systematic review and NMA protocol, our study is limited by the quality and consistency of the data extracted from the included studies. We took steps to mitigate these issues by using a rigorous assessment of the RoB and data extraction processes. In addition, the statistical and moderation analyses we have planned will allow us to explore the sources of heterogeneity and potential moderators of treatment effects. Finally, we have adhered to ethical guidelines and plan to disseminate our findings to relevant stakeholders to facilitate the translation of research into practice.

### Conclusions

Despite these limitations, we believe that our study provides valuable insights into the efficacy of PAIS for improving cognitive performance in children with ASD. Our findings suggest that clinicians and policy makers should consider incorporating PAIS into interventions aimed at improving cognitive function in this population. Furthermore, our study highlights the need for additional research in this area, including larger, well-controlled studies to further examine the comparative effectiveness of different types of PAIS. Overall, we hope that our study will contribute to the development of evidence-based interventions that can help improve outcomes for children with ASD.

## Appendix

Multimedia Appendix 1 Cognitive measures in effect calculation.

Multimedia Appendix 2 PRISMA-NMA (Preferred Reporting Items for Systematic Reviews and Meta-Analyses Protocols for Network Meta-Analyses) checklist.

## Abbreviations

ASD: autism spectrum disorder
EF: executive function
MeSH: Medical Subject Headings
NMA: network meta-analysis
PAIS: physical activity intervention strategies
PICOS: Population, Intervention, Comparison, Outcome Measures, and Design
PRISMA: Preferred Reporting Items for Systematic Reviews and Meta-Analyses
PRISMA-NMA: Preferred Reporting Items for Systematic Reviews and Meta-Analyses Protocols for Network Meta-Analyses
RCT: randomized controlled trial
RoB: risk of bias
SMD: standardized mean difference

## Data Availability

This is not applicable at present, as this is a protocol paper. However, future and final data sets will be available from the corresponding author upon reasonable request.
