# Supplementary material for: Physical Activity Interventions for Improving Cognitive Functions in Children With Autism Spectrum Disorder: Protocol for a Network Meta-Analysis of Randomized Controlled Trials
Source: JMIR Res Protoc. 2023 Jun 28;12:e40383. doi: 10.2196/40383 (PMC10365605; doi:10.2196/40383)
Supplement: Multimedia Appendix 1 [file resprot_v12i1e40383_app1.docx]

**Supplementary**

**Table of Cognitive Measures in Effect Calculation**

|  | **Memory** | **Attention and**  **processing speed** | **Executive function** |
| --- | --- | --- | --- |
| **Measures** | Story recall,  verbal list learning,  Design Learning Test,  Rey Complex Figure Test,  Factor-analytically derived memory score from test battery,  Hopkins Verbal Learning Test,  WMS, TOMAL  Rey Auditory Verbal Learning Test, HKLLT, WRAML | WISC-IV Processing Speed Index,  Grooved Pegboard Test,  Finger Tapping Test,  Motor,  Continuous Performance Test,  RAD,  Arsenow’s test,  Trail Making Test A,  Brief Test of Attention, WIAT-III | BRIEF, Trail Making Test B,  Verbal and nonverbal fluency,  WMS Visual reproduction,  Digit and visual span,  WCST Perseverative Errors,  Average of reasoning and working memory standardized domain scores,  Visuospatial Working Memory test,  Letter number sequencing,  Digit sequencing,  Mazes subtest,  Tower of London,  WAIS-III Backward digits, CBTT, CTT, DSFBT; FPT; GNG |

*Note*. WIAT-III: Wechsler Individual Achievement Test-Third Edition; WMS = Wechsler Memory Scale; WISC-IV = Wechsler Intelligence Scales for Children-IV; RAD = Redundancy-Associated Deficit; WCST = Wisconsin Card Sorting Test; BRIEF: Behavior Rating Inventory of Executive Function; CBTT: Corsi block-tapping task; CTT: color trails test; DSFBT: digit span forward and backward test; FPT: Five-Point Test; GNG: go-no-go task; HKLLT: Hong Kong List Learning Test; TOMAL = Test of Memory and Learning; WRAML = Wide Range Assessment of Memory and Learning; Cognitive measurements were categorized according to Désaméricq et al. (2014).
